# Clinical endpoints special session, ISCEV 2025, Utrecht

**DOI:** 10.1007/s10633-026-10108-8

**Published:** 2026-05-20

**Authors:** R. Hamilton, D. Birch, K. Stingl, K. Fujinami, K. Holopigian, C. J. F. Boon, J. Moseley, J. J. McAnany, A. V. Cideciyan, M. M. van Genderen

**Affiliations:** 1 Department of Clinical Physics & Bioengineering, Royal Hospital for Children, Glasgow, UK; 2 College of Medicine, Veterinary & Life Sciences, University of Glasgow, Glasgow, UK; 3 Retina Foundation of the Southwest, Dallas, USA; 4 University Eye Hospital, Center for Ophthalmology, University of Tübingen, Elfriede-Aulhorn-Straße 7, 72076 Tübingen, Germany; 5 Laboratory of Visual Physiology, Division of Vision Research, National Institute of Sensory Organs, National Hospital Organization Tokyo Medical Center, Tokyo, Japan; 6 University College London Institute of Ophthalmology, London, UK; 7 Moorfields Eye Hospital, London, UK; 8 Department of Ophthalmology, Keio University School of Medicine, Tokyo, Japan; 9 Department of Biological Sciences, Graduate School of Science, The University of Tokyo, Tokyo, Japan; 10 Novartis Pharmaceuticals Corporation, East Hanover, USA; 11 Amsterdam University Medical Center, Amsterdam, The Netherlands; 12 Leiden University Medical Center, Leiden, The Netherlands; 13 European Medicines Agency, Amsterdam, The Netherlands; 14 Department of Ophthalmology and Visual Sciences, University of Illinois Chicago, Chicago, USA; 15 Department of Biomedical Engineering, University of Illinois Chicago, Chicago, IL USA; 16 Center for Hereditary Retinal Degenerations, Scheie Eye Institute, University of Pennsylvania, Philadelphia, USA; 17 Bartiméus Diagnostic Center for Complex Visual Disorders, Zeist, The Netherlands; 18 University Medical Center Utrecht, Utrecht, The Netherlands

**Keywords:** Retinal dystrophies, Clinical endpoints, Clinical trials, Patients perspective

## Abstract

This report summarises the Clinical Endpoints Special Session held during the 62nd annual symposium of the International Society for Clinical Electrophysiology of Vision (ISCEV), convened at Tivoli Vredenburg, Utrecht, The Netherlands. The session brought together clinicians, regulators, industry representatives, and a patient voice to consider the state of clinical endpoints in trials for inherited retinal disorders (IRDs). Discussions covered the adequacy of current endpoints, challenges of disease heterogeneity, regulatory expectations, operational feasibility, and the perspectives of patients. The session highlighted the need to anchor emerging endpoints to clinically meaningful outcomes and to minimise assessment burdens.

## Introduction

The increase in new therapies with potential to restore vision in inherited retinal disorders (IRDs) was triggered by the success of voretigene neparvovec-rzyl (Luxturna®) for *RPE65*-associated IRD [1]. Dozens more trials of gene therapies, optogenetics, pharmacological approaches and regenerative medicine are underway [2]. This rapid growth has challenged the array of biomarkers and clinical endpoints accepted by medical regulators such as the Food and Drug Administration (FDA) in the USA and the European Medicines Agency (EMA) [3–5]. Well-established primary endpoints such as visual acuity or visual field improvements [6, 7] may not reflect improvements, or prevention of loss, in some IRD therapy trials [2]: at least part of Luxturna’s success was FDA approval for the novel use of the standardised multi-luminance mobility test (MLMT®) as its primary endpoint [8]. Rate of photoreceptor loss, established via imaging, has recently been found to be acceptable by the FDA as a primary endpoint [9, 10]. Clinicians, industry sponsors, patient representatives and regulators alike recognise and encourage the need for approval of new endpoints [11–16].

The International Society for Clinical Electrophysiology of Vision (ISCEV) has developed and updated standards for visual electrophysiology since 1989 [17–21], and more recently collaborated with the Imaging and Perimetry Society to develop a clinical guideline for the full-field stimulus test (FST) [22]: further collaborations for clinical guidelines are underway for nystagmus assessment and full-field pupillography. These documents underpin both clinical practice and research design: where validated endpoints are identified, the need for their standardisation has been established as a priority [23]. Endpoints using standardised tests are appealing because adherence to the standard helps to ensure well-defined and reliable measures [24, 25]. As new treatments reach clinical trials, further research into new endpoints, paradigms and protocols will be needed.

The Utrecht session sought to take an international and inclusive look at the challenges facing IRD clinical trials, and at possible solutions via existing and emerging endpoints and alternate strategies. The five themes which emerged from the special session are explored in turn below.

## The desirability and inadequacy of current endpoints

The ideal properties of an endpoint were explored by several speakers, noting dependence on trial phase and on the therapeutic expectation (improvement *versus* stabilisation or slowed progression). Both the clinical relevance (associated with a meaningful aspect of the patient's perception of benefit) and the measurement properties (accuracy, reliability, sensitivity to detect change, minimum clinically important difference (MCID)) of the endpoint should be well understood. Endpoints should be able to discriminate between health and disease or disease stages (e.g. Table 1, [26]). The endpoint must be achievable from an operational perspective: multi-site studies require adequately similar equipment on each site with training and even certification of staff undertaking testing, and sometimes central reading or interpretation facilities.

It was recognised that best corrected visual acuity (BCVA) may not be the most appropriate primary endpoint in some IRDs, for example where central vision is affected very late in the disease progression: primary endpoint choice needs to be appropriate for the condition. Secondary endpoints are intended to support primary findings and/or provide additional information and cannot be adopted as proof of efficacy in a clinical trial which has failed on its primary endpoint. The statistical hypotheses to be tested and the order of testing should be pre-specified. The primary analysis of the primary variable should be distinguished from supporting analyses in a way which guards against the risk of false positive findings from multiple statistical tests and a post hoc choice of the most favourable results from multiple analyses [27, 28]. The need for appropriate primary endpoints [29] in IRD clinical trials was widely acknowledged in the session: meeting this need was important in order to bring effective therapies to market, thereby improving patient outcomes. Inappropriate endpoints waste time and money—a drug can take US$1.6–2 billion and 10–15 years to reach the market—as well as the unethical burden to patients enrolled in trials which selected a suboptimal primary endpoint [12].

While surrogate endpoints—such as those reflecting a structural or anatomical measure—may be acceptable at marketing authorisation if fully validated, the additional burden of robust validation in longitudinal datasets can be challenging in rare and slowly progressing disease settings.

## Challenges beyond the endpoints

The session reflected the challenges of disease heterogeneity in IRDs—even within a single genotypic group, such as *USH2A*-related disease, patients present with diverse symptom combinations and rates of progression. IRDs are generally rare, meaning trials draw on small cohorts: combined with genotypic and/or phenotypic heterogeneity, this makes it unrealistic to establish small effects using group statistics. Different therapeutic expectations (improvement versus stabilisation or slowed progression) may require different assessment strategies. Demonstrating any slowing of progression requires trials which span many years and can maintain consistent measurement techniques. Genetic and/or ethnic variations may preclude generalisability of trial findings from a particular population. Operational feasibility can be a limiting factor on the choice of study design and endpoints, and feasibility of infants or young children completing a particular test can be a barrier to recruitment in a primary analysis population for a trial, regardless of whether age-appropriate tests are developed.

## Regulatory aspects

Over 30 high-quality regulatory authorities operate worldwide. In this session, speakers discussed interactions with the FDA and the Pharmaceuticals and Medical Devices Agency (PMDA), the Japanese regulatory agency, and presented on behalf of the EMA. The close involvement of the FDA in shaping the RUSH2A Extension, which aims to quantify the clinical relevance of the FST, was described.

In Japan, five phase 3 trials for IRDs were discussed, including Luxturna® for *RPE65*-associated IRD [30]. Given the predominance (68%) of subjects from the White population reported worldwide, and following discussions with the PMDA, it was agreed that a phase 3 trial was indicated to investigate efficacy and safety in Japanese patients, in whom disease-causing variants of RPE65-retinopathy may differ from those in other ethnic groups [30]. In 2017, the device manufactured by Diagnosys LLC and used to perform the FST was approved as a medical device in Japan and the standardised use of the FST procedure was approved by the Japanese PMDA as a primary endpoint. Results from four Japanese patients enabled approval in 2023 [30] and since July 2024, Luxturna® for *RPE65*-associated IRD is an insurance-covered treatment in Japan, with a mandatory requirement to collect up to five years of safety and efficacy data in all patients treated within the five years following regulatory approval.

From the EMA perspective, the framework for the licencing of medicinal products for human use is determined by European Union (EU) legislation [31], guidance from the International Council for Harmonisation of Technical Requirements for Pharmaceuticals for Human Use (ICH) and EU-specific rules and guidelines. This framework sets the standards for approval of new medicinal products. During a marketing authorisation assessment (MAA), the evidence of efficacy, or benefits, of the medicines has to be weighed against its safety issues and be found positive. This is why the choice of primary endpoint is of fundamental importance as it furnishes the primary evidence of benefit. The variable chosen as the primary endpoint should be capable of providing the most clinically relevant and convincing evidence directly related to the primary objective of the trial. The selection of the primary variable should reflect the accepted norms and standards in the relevant field of research. The use of a reliable and validated variable with which experience has been gained either in earlier studies or in published literature is recommended. There should be sufficient evidence that the primary variable can provide a valid and reliable measure of some clinically relevant and important treatment benefit in the patient population described by the inclusion and exclusion criteria. The primary endpoints selected for confirmatory studies should be clinically relevant and reflect disease burden or be of adequate surrogacy for predicting disease burden or sequelae [32, 33]. Evidence of durability of response is also required: however, in a slowly progressive condition, two year trial data may need to be submitted at MAA with further long term data collected post marketing. The timing of data collection and primary analysis should be informed by the exploratory trial data in the condition and the specific natural history of the disease progression. The importance of understanding the clinical significance of the primary endpoint via anchoring to outcomes which are meaningful to the patient, such as performance tests, patient reported outcomes (PROs) or years of quality vision gained was emphasised. Stabilisation of function as an outcome was noted to be important: a definition, including the duration of follow-up required to establish stability, would be required by the EMA. Further proposed outcomes have included visual sensitivity assessed by perimetry and microperimetry and mobility testing: again, the key to regulatory approval lay in detailed understanding of the test and tailoring to the target population.

Surrogate endpoints based on structural or anatomical findings must have their surrogacy rigorously established by understanding the biological relationship of surrogate and clinical outcome as well as the mechanisms of action of the intervention in the condition. The intervention-driven change in the surrogate endpoint should predict change in a clinical efficacy endpoint such as symptoms or a functional outcome. Multiple trials are generally required to establish surrogacy (exploratory, confirmatory, trial level) and to address concerns that the surrogate may not be a true predictor of the clinical outcome of interest [5, 34]. It was noted from the floor and the panel that surrogate endpoints are most useful in early phase I/II trials when a drug’s functional target has yet to be completely understood.

Regulators may not arrive at the same conclusions regarding a particular endpoint. For example, two geographic atrophy (a form of advanced dry age-related macular degeneration) therapies, Syfovre® & Izervay™ were approved by the FDA based on a structural outcome, lesion size on fundus autofluoresence [35], but rejected by the EMA, which considered that slowed lesion growth did not lead to clinically meaningful benefits for patients [36, 37].

## The patient’s voice

Patient representative Casper van Dijk, 28 years old, first experienced visual difficulties around the age of 16 when he realised cycling at night was becoming difficult. Following “a lot of tests”, he was diagnosed with X-linked retinitis pigmentosa due to variants in *RPGR*. As an adult, Casper was enrolled on an *RPGR* gene therapy trial in 2024 and was treated in both eyes. He told the session that the decision to enrol was difficult but he decided that the possibility of stopping disease progression outweighed the risks. He described the weeks following treatment as scary and stressful. In this early period following surgery, his vision was less than before the operation and he was unsure if the surgery had been successful. Also during this time, he was required to visit the clinic for extensive follow-up testing. He described this period as having a lot of uncertainty: Did the surgery work? Will my vision improve? Will improvements be permanent?

After about a month, Casper was able to see as well as prior to treatment. More follow-up testing was required, which he acknowledged he felt to be a burden. The amount and burden of testing that was required was emphasised throughout the discussion, including the number of test days, travel time and expense as well as how onerous testing could be. Sponsors do consider this issue in the design of the trial and discuss the extent of the burden with prospective subjects. Casper said that although each test was doable, the visit was tiring and more rest between tests could have made it more tolerable. When asked which test was most relevant to characterise his visual experience, he was unsure. He said he found maze navigating the most enjoyable, as it differed considerably from the other tests. When asked what he would consider a successful outcome, he replied that keeping his current level of vision for the remainder of his life would be a success to him. Conversely, if treatment were ultimately not successful, he would continue his life with the knowledge that he would eventually become blind. Overall, he said he would not change his decision to participate in the trial.

The interview ended by asking if he had advice for others who are considering enrolling a trial. He responded that his experience has been good, but he has routinely attended clinic for extensive testing for years, so he is very familiar with what will happen and what is expected. He also noted that discussion with the staff, technicians and physicians is very important to develop a personal connection with the team.

This valued interview highlighted the burden of repeated assessments—especially if lengthy participation is required in therapy trials for slowly progressing diseases. An additional burden may arise from the emotional strain of uncertainty after receiving a novel therapy. For many patients, stabilisation of vision is a valued outcome.

The critical role of the patient in determining outcomes was emphasised from the industry perspective, where patient advisory boards are used to understand how diseases impact the patient and their caregivers. This helps to shape the outcome measures which might capture changes which would be meaningful to the individual.

Validated PROs, such as the Michigan Retinal Degeneration Questionnaire (MRDQ), can capture functional domains which matter to patients. While results may correlate with objective measures including FST [38] and (micro)perimetry [39], correlation alone is not enough: regulators need to know how much change in an endpoint such as the FST is meaningful to patients, requiring anchor-based methods.

## The way forward

### Dialogue with regulatory bodies

Regulators in the USA (FDA) and in Japan (PMDA) are open to dialogue about endpoint selection and development. The EMA also has processes to provide advice about the most appropriate way to generate robust evidence on a medicine's benefits and risks [40], and development of new methodologies such as novel biomarkers [41]. International guidelines encourage early engagement with regulatory authorities, particularly when a study has novel endpoints which are considered critical to the study quality [32, 42].

Several international collaborations are working on accelerating IRD treatments: one is the Regulatory Endpoints and Trial Design for IRDs (REDI) working group established by the Foundation Fighting Blindness Consortium in the USA. This group aims to identify the best functional and structural measures and to collaborate with industry and regulators to propose best endpoints for IRD clinical trials. Their exemplar RUSH2A study described the natural history of *USH2A*-related retinal degeneration, and also evaluated suitability of measures for clinical trials, identifying the FST [22] to have high quality measurement properties [26] in addition to being sensitive, relatively quick to administer, and with limited floor effects. FDA input helped direct the current RUSH2A extension which aims to quantify the FST MCID, and to quantify how much change of a clinically meaningful ‘anchor’ is predicted by a specified FST change [43]. Candidate anchor tests include the MLMT® or a validated PRO assessment.

### Inclusion of the patient voice

Greater integration of the patient voice into trial design was identified as a priority, both ethically and scientifically, as also indicated in international guidelines [32, 43].

### Promising novel endpoints

In addition to the FST, microperimetry, low luminance visual acuity and the MRDQ, several other potential endpoints were discussed. For example, a phase 1/2 gene therapy trial in patients with *RLBP1* retinal dystrophy with severely delayed dark adaptation chose a dark-adapted retinal sensitivity test as its primary endpoint [44] and dark-adapted dynamics has shown evidence of detailed, localised rescue of rod function after therapy [45]. The electrically-evoked response (EER), which excites non-functioning photoreceptors and induces a “flash” visual evoked potential (VEP) [30] may prove helpful for monitoring disease in patients with advanced disease. Virtual-reality based mobility tests may provide a lower cost alternative to the manufacturer-specific MLMT® [8]. Flavoprotein fluorescence imaging was discussed as a biomarker which showed evidence of correlating with mitochondrial stress and which may offer early signals of slowed degeneration before structural changes are evident [46]. These are included here as discussed in the session but do not constitute a regulatory recognition.

### Promising approaches

Individualised evaluation rather than group effects may benefit therapeutic development for rare diseases and heterogeneous phenotypes [47]. Where patient populations are very limited in number and conventional statistical significance is difficult to achieve, there is EMA guidance about exceptional circumstances and small populations [48, 49]. If the aim is to stop or slow progression, longer-term follow-up could be effective. Anchor-based analyses to define MCIDs are key and require understanding measurement variability and correlation. For example, the Test–Retest Reliability in Patients with Inherited Retinal Dystrophies (REPEAT) study has quantified the reliability of the FST [38], as well as, importantly, its correlation with the MRDQ [39]], key foundational work for anchoring. Composite indices integrating structural data, functional data, and PROs were also highlighted as potential ways forward.

## Conclusion

The development of innovative treatments for IRDs predominantly affecting vision in dim ambient light conditions driven by rod function presents a challenge for currently recognised outcome measures in clinical trials. The patient voice reminds us that preservation or improvement of rod-mediated as well as cone-mediated vision can both be hoped-for outcomes. Multiple tests, such as the FST and others, may be better primary endpoints than those currently accepted by the regulators. It is in the best interests of patients for sponsors to engage early with regulatory bodies and other stakeholders such as researchers and clinicians to optimise trial design and maximise the chance of bringing effective therapeutics to market. The ISCEV annual meeting in Utrecht presented an opportune setting to discuss these issues.

## Data Availability

No datasets were generated or analysed during the current study.

## Abbreviations

BCVA: Best corrected visual acuity
EER: Electrically-evoked response
EMA: European medicines agency
EU: European Union
FDA: Food and drug administration
FST: Full-field stimulus test
ICH: International Council for Harmonisation of Technical Requirements for Pharmaceuticals for Human Use
IRD: Inherited retinal disorder
ISCEV: International Society for Clinical Electrophysiology of Vision
MAA: Marketing authorisation assessment
MCID: Minimum clinically important difference
MLMT®: Multi-luminance mobility test
MRDQ: Michigan retinal degeneration questionnaire
PMDA: Pharmaceuticals and Medical Devices Agency (Japan)
PRO: Patient reported outcomes
REDI: Regulatory endpoints and trial design for IRDs
VEP: Visual evoked potential

## Declarations

### Statement on the welfare of animals

The study involved no research on animals.
